# Dermoscopic rainbow pattern in malignant blue nevus^؆^

**DOI:** 10.1016/j.abd.2025.501273

**Published:** 2026-01-23

**Authors:** Ana Luiza Mapurunga Gonçalves, Elisa Scandiuzzi Maciel, Isadora Ferreira da Fonseca, Milvia Maria Simões e Silva Enokihara, Sérgio Henrique Hirata

**Affiliations:** a Department of Dermatology, Escola Paulista de Medicina, Universidade Federal de São Paulo, São Paulo, SP, Brazil; b Department of Pathology, Escola Paulista de Medicina, Universidade Federal de São Paulo, São Paulo, SP, Brazil; c Postgraduate Program in Translational Medicine, Department of Medicine, Escola Paulista de Medicina, Universidade Federal de São Paulo, São Paulo, SP, Brazil

Dear Editor,

A 74-year-old female patient presented with a 5-cm diameter grayish-blue nodule on her left buttock (Fig. 1). Dermoscopy revealed a bluish background, rainbow pattern, and bright white structures surrounding an ulcerated area. Polymorphic vessels and white, black, and red coloration were observed. The patient reported that the lesion had been present since birth, with accelerated growth over the past two years (Fig. 2). Histopathological examination revealed mitotic figures, including atypical mitoses, and involvement of nerve fibers in the deep reticular dermis (Fig. 3). Intense cellularity was observed, represented by the proliferation of atypical dendritic melanocytes, marked nuclear polymorphism, and hyperchromasia (Fig. 4).

**Fig. 1:**
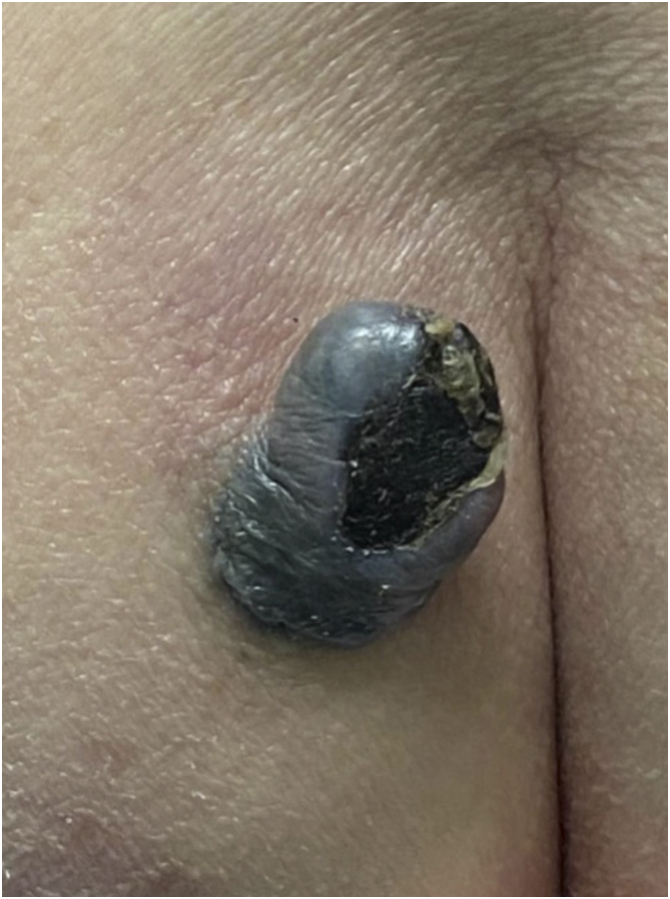
Exophytic nodule, bluish in color, with central ulceration and a discrete area of ​​irregular, blackened spot at the base, measuring approximately 5 cm in diameter at its longest axis, located on the left buttock. Erythema is also observed at the upper left pole of the lesion.

**Fig. 2:**
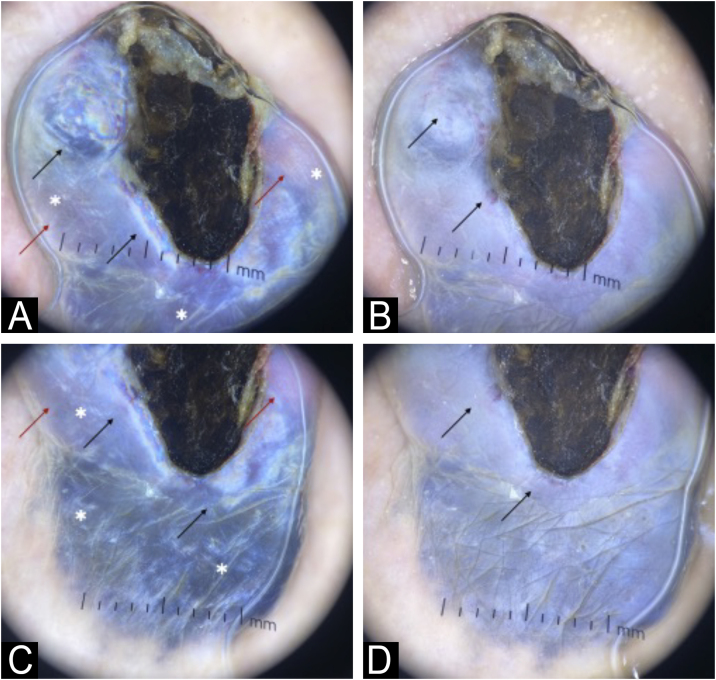
(A) Dermoscopy (×10) with polarized light: Upper pole of the lesion showing a diffuse, exuberant rainbow pattern (black arrow) over a bluish area interspersed with reddish-white areas (red arrow). Bright white structures (white asterisk) are present throughout the region. A serohematic structure is observed in the center. (B) Dermoscopy (×10) with immersion, non-polarized light: Upper pole of the lesion showing a bluish area, with evident polymorphic vessels (black arrow). (C) Dermoscopy (×10) with polarized light: Lower pole of the lesion showing a diffuse, exuberant rainbow pattern (black arrow) over a bluish area interspersed with reddish-white areas (red arrow). Bright white structures (white asterisk) are present throughout the region. A serohematic structure is observed in the center. A poorly defined and irregular area is observed in the most distal portion. (D) Dermoscopy (×10) with immersion, non-polarized light: Lower pole of the lesion showing a bluish area, with polymorphic vessels (black arrow) and in the most distal portion of the lesion, a poorly defined and irregular area.

**Fig. 3:**
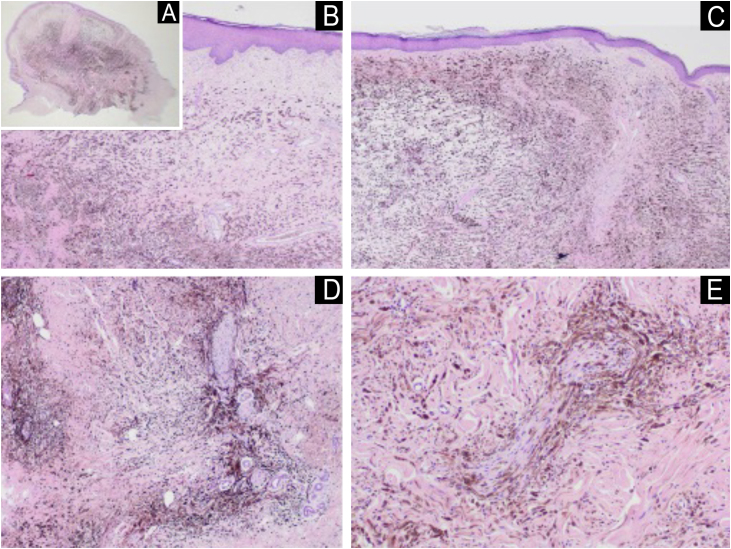
(A) Panoramic view of the lesion showing its polypoid aspect. (B and C) They show diffuse proliferation of isolated dendritic melanocytes, distributed in an isolated manner among collagen fibers or forming bundles, occupying the entire dermis, without compromising the epidermis and with a compromised deep margin. (D) Proliferation of melanocytes involving cutaneous appendages, diffuse proliferation of isolated dendritic melanocytes, distributed in an isolated manner or forming bundles, without compromising the epidermis and with a compromised deep margin (Hematoxylin & eosin, ×20). (E) This melanocytic proliferation compromises nerve fibers of the deep reticular dermis (Hematoxylin & eosin, ×20).

**Fig. 4:**
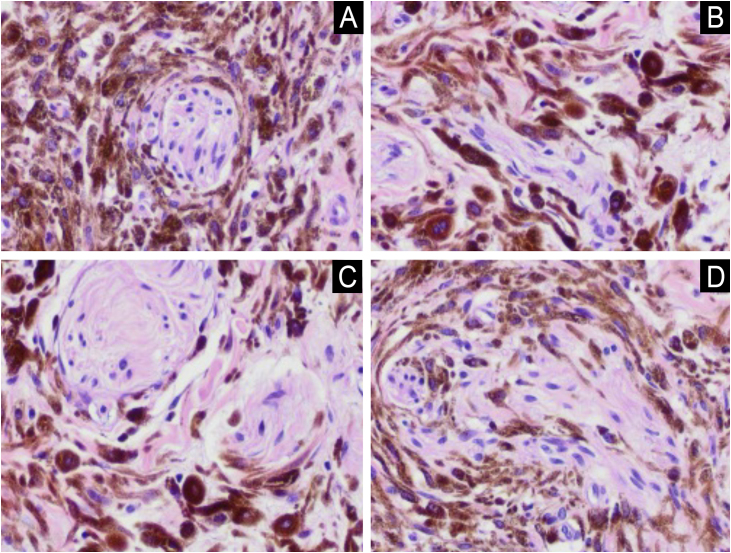
In detail, atypical dendritic melanocytes are noted, with a marked degree of nuclear polymorphism and hyperchromasia, and infiltrating nerve fibers in Figures A, B, C and D (Hematoxylin & eosin, ×100).

The term “malignant blue nevus” (MBN) refers to a rare and aggressive subtype of melanoma described by Allen and Spitz in 1953. This type of melanoma can originate from a pre-existing blue nevus (BN), particularly a cellular BN, although less frequently it can arise from a common BN. In addition, the MBN can manifest *de novo*, with an appearance similar to a BN.^1^

MBN occurs in typical BN areas: scalp, face, and buttocks. It is more common in the elderly, with a predominance in males. Clinically, it presents as blue or black nodules or plaques and may ulcerate as it evolves. Late diagnosis is a determining factor for the aggressive behavior of MBN, which translates into a greater likelihood of metastasis and mortality.^2^, ^3^

In a retrospective epidemiological study on MBN conducted in 2023, Yumeen et al. reported this aggressive behavior with a predisposition to metastasis, especially to lymph nodes and lungs. This finding corroborates initial studies of the tumor but diverges from the results of a case series published in 2009, which found no significant differences in the behavior of MBN and melanoma.^3^, ^4^

Although the dermoscopic findings of MBN are not widely described in the literature, it shares characteristics of melanoma and BN. This makes dermoscopic differentiation from pigmented lesions challenging.^1^ The presence of bright white lines, polymorphic vessels, asymmetrical distribution of blue coloration, bluish globules at the lesion periphery, gray color, blue-gray veil, multiple colors, and chaotic pattern (which may reflect asymmetry in structures or color) is indicative of malignancy.^5^, ^6^

The “blue and black” rule described by Argenziano et al., originally formulated to facilitate the diagnosis of nodular melanoma, states that the presence of both colors in at least 10% of the lesion extent has a positive predictive value of 90% for malignancy. The clinical case described, in addition to fitting the “blue and black” rule, shows polymorphic vessels, which strengthens the suspicion of malignancy.^7^

The histopathological correlate of the rainbow pattern on dermoscopy is not yet fully elucidated. It is suggested that it is related to the heterogeneity of the dermal layers, which interfere with the absorption, reflection and transmission of polarized light, resulting in the observed color spectrum.^8^, ^9^, ^10^ In cases such as the present one, intense cellularity may contribute to this phenomenon.

The rainbow pattern was initially described in Kaposi's sarcoma, characterized by the presence of multiple colors visible under polarized light dermoscopy. Its origin is attributed to the phenomenon of light diffraction, that is, the ability of waves to bend around obstacles. However, this finding has been identified in several other conditions, such as stasis dermatitis, lichen planus, hemosiderotic dermatofibroma, blue nevus, and melanoma. Although it may be present in invasive melanomas, the rainbow pattern, alone, is not indicative of malignancy. However, when associated with specific dermoscopic criteria, it may suggest tumor invasion.^8^, ^9^, ^10^

The most recent theory proposes that the rainbow pattern observed in dermoscopy is related to the phenomenon of dichroism. Unlike diffraction, dichroism is the property of certain substances to absorb polarized light differently depending on the direction of incidence. This results in different colors depending on the angle of observation. In dermoscopy, this effect can occur in structures with heterogeneous organization and specific orientation, which interact unevenly with polarized light, generating the multicolored spectrum characteristic of the rainbow pattern.^9^

The history of congenital lesions suggests that the MBN arose as a malignant transformation of a previous BN. The case described illustrates an unusual dermoscopic finding, reinforcing the importance of thorough evaluation of pigmented nodular lesions.

## ORCID IDs

Elisa Scandiuzzi Maciel: 0000-0003-4322-1260

Isadora Ferreira da Fonseca: 0009-0008-3821-1176

Milvia Maria Simões e Silva Enokihara: 0000-0002-3340-4074

Sérgio Henrique Hirata: 0000-0003-4026-9664

## Authors' contributions

Ana Luiza Mapurunga Gonçalves: Design and planning of the study; data curation; methodology; writing – original draft; writing – review and editing; approval of the final version of the manuscript.

Elisa Scandiuzzi Maciel: Data curation; methodology; approval of the final version of the manuscript.

Isadora Ferreira da Fonseca: Data curation; methodology; approval of the final version of the manuscript.

Milvia Maria Simões e Silva Enokihara: Data curation; writing – original draft; writing – review and editing; supervision; methodology; approval of the final version of the manuscript.

Sérgio Henrique Hirata: Data curation; writing – original draft; writing – review and editing; supervision; methodology; approval of the final version of the manuscript.

## Financial support

None declared.

## Research data availability

Not applicable.

## Conflicts of interest

None declared.
